# Louisa Warnes, 1836-1914

**Published:** 1914-10-10

**Authors:** 


### SOME WORKERS OF NOTE.

### Louisa Warnes, 1836-1914.

Norfolk has good cause to be proud of its famous women. Elizabeth Fry was born in Norwich in 1780, and owed much of her success in life to the training she received under the guidance of her father, John Gurney, a member of the Society of Friends, of which Society she was ultimately acknowledged by her co-religionists as a minister. Elizabeth Fry was a philanthropist who, next to Howard, was the chief promoter of prison reform in Europe, to which object she devoted most of her immense and manifold energies. In 1827 British hospitals and asylums for the insane were classed as houses of detention, a fact which may surprise many hospital workers of to-day. It clearly indicates the enormous developments in the organisation and administration of our hospitals, due to the increasing knowledge, the reforms in system, the developments of science, and the revolution in treatment which the growth of civilisation has brought about during the last eighty years.

Another Norfolk woman who has worked for upwards of fifty years in the city of Norwich, Louisa Warnes, passed peacefully into the presence of Almighty God on the 22nd ultimo. She was the eldest daughter of John Warnes, of Bolwick Hall, Marsham, the author of "Warnes on the Flax Crop," which excited great interest and was fruitful in developments in connection with the Great Exhibition in 1851. As the eldest daughter, owing to her father's death at the commencement of her womanhood, Louisa Warnes became the moving spirit in her family. She exercised a splendid influence over her five sisters, and did her utmost to train, guide, and help her two brothers. She had been carefully educated and had developed unusual facilities in the acquirement of languages, including French, German, Italian, Hebrew, and Greek. The two latter languages she learnt with the main object of enabling her to study original documents and authorities, so that she might have the advantage of a personal knowledge of the text on which the New and Old Testaments rest. When still a girl she gave many indications of her deep piety, the benevolence of her disposition, her strength of purpose, and the clearness and independence of her judgment. The beauty of her character, her unselfishness and liberality, her life devoted to the service of God and for the benefit of mankind, were as remarkable as they were fruitful in results. She had extraordinary power and influence over the young of both sexes, and Canon Aitken on the day of her funeral, from an intimate knowledge of and long association in Norwich with Louisa Warnes during her fifty-two years' work in that city, testified that it is no exaggeration to say that thousands have reason to call her blessed for the ministry of love which God enabled her to perform towards them, It is remarkable, too, that those who touched her life remained under the beneficent influence which she exercised over them for the rest of their lives. Louisa Warnes was gifted in many ways, and it was her joy to impart knowledge to those who came in contact with her. Her happiest hours were those when, surrounded by learners, she was enabled to make clear to them some of the great works of Italy, France, Germany, and of other foreign countries with whose languages she was acquainted. She possessed the capacity for easy and graceful speech, which rendered her public utterances always attractive. Canon Aitken testified that her quiet manner would quickly still a multitude of noisy factory girls in a way which few others could do. She was a fine elocutionist, and the boys at the Norwich Grammar School owed a great debt of gratitude to her, for by her training they were enabled to take the first place in elocution in competition with others of a similar age. She had wonderful tenderness, quickness of sympathy, and unfailing love, characteristics which made her relation to others of the highest value.

It will interest our readers to know that she was the leading spirit who, in conjunction with Dr. and Miss Gibson, founded the Norfolk and Norwich Staff of Nurses, originally known as the Bethel Street Nurses, so long ago as the year 1866. At that period in East Anglia trained nurses were unknown, but Louisa Warnes foresaw the need and attracted many good women willing to be trained as nurses at the hospitals. They constituted the first staff of trained nurses for the sick and poor in Norwich and its neighbourhood. Nurses have never had a truer or wiser friend than Louisa Warnes. The last day of her active life she spent at 32 Surrey Street, first at a committee meeting and then with those of the nursing staff who were present in the Home. The affection between Miss Warnes and the nurses was deep and true, and in her they had a very real friend. As the years passed and great changes took place in the organisation and training of nurses, the District Nursing Association was instituted in Norwich, which won for her many new friends and did immense good in the homes of the people. At her funeral the number of those who appreciated her work, who owed her gratitude for the influence she had exercised upon their life, and whose love she had won, was so numerous that it was not possible for all of them to find a place at the service at St. Augustine's Church. Some hundreds walked the long distance to the cemetery, where the scene was remarkable. All who can appreciate the power of one who throughout her life walked with God will best realise the void caused by her death, which the mourners felt could never be filled on this side the grave. We have felt that the great work for nurses, for the sick, for the humble, for the needy and the suffering, which Louisa Warnes accomplished during her life should be gratefully recorded in the columns of The Hospital.

